# The riddle of the Sphinx: why sphingosine‐1‐phosphate may help define molecular mechanisms underlying risk stratification for serious COVID‐19 infections

**DOI:** 10.15252/emmm.202013533

**Published:** 2020-12-16

**Authors:** Hugh Rosen, Michael B A Oldstone

**Affiliations:** ^1^ The Scripps Research Institute La Jolla CA USA

**Keywords:** Biomarkers & Diagnostic Imaging, Microbiology, Virology & Host Pathogen Interaction

## Abstract

The sphingosine‐1‐phosphate (S1P) is a lysophospholipid signaling molecule with important functions in many physiological and pathological conditions, including viral infection. In this issue of *EMBO Molecular Medicine*, Marfia *et al* present a risk stratification based on S1P serum level as a novel prognostic indicator for COVID‐19 severity.

## Graphical abstract

H. Rosen and M. B. A. Oldstone discuss the importance of sphingosine‐1‐phosphate serum level as a novel severity predictor for COVID‐19 identified by G. Marfia and colleagues, in this issue of *EMBO Mol Med*.

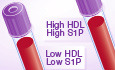

Jacob Bronowski, in his intellectual history of science, "The Ascent of Man" mused upon the simple, impertinent question that unlocks the most pertinent of answers (Bronowski & British Broadcasting Corporation, 1973). Pandemic viral pathogens ask the most difficult questions of humankind. Harsh and rapid selective pressures at the level of populations, from a facile and rapidly adapting viral genome, identify the gaps in our host defenses and homeostatic control. These poor outcomes, framing the challenges of risk stratification and adaptive clinical management of the COVID‐19 pandemic, have been especially problematic. Globalism and significant intercontinental travel are even more profound mechanisms today for rapid pathogen spread (Oldstone, 2020). The pressures of this new pandemic suggest that that a societal lack of historic and scientific literacy even among some scientists can lead to a baying fear of contagion; such fear is amplified by unconstrained mathematical modeling and tabloid sensationalism. The antidote to these unfortunate excesses is deliberate, quantitative molecular medicine in which hypothesis‐driven data collection defines contributing factors to patient outcome. These factors in turn generate new testable hypotheses and mechanisms that lead to adaptive improvements in patient care.

The article in this issue of *EMBO Molecular Medicine* by Marfia and colleagues (Marfia *et al*, 2021) asks such a simple and impertinent question. They suggest a potential mechanistic linkage between a serum measurement of sphingosine‐1‐phosphate (S1P) and the progression to and outcome of the serious inflammatory phase of COVID‐19 infection. Mortality data from Europe and the United States highlight the role of co‐morbidities in COVID‐19 mortality, with ~95% of deaths associated with key underlying risk factors including age, dementia, diabetes, cardiovascular disease, among others. However, even in defined risk groups such as patients in skilled nursing facilities, individual variations in progression to inflammatory phase and poor outcomes are yet to be understood. This study provides a signpost to a potential explanation.

S1P was named for its riddle‐like physical properties and pleiotropic biological effects (Rosen *et al*, 2013). It is a lysophospholipid signaling molecule, a problematic hydrophobic strong zwitterion with 2 negative and 1 positive charges at physiological pH that is essentially insoluble in aqueous solution unless bound to a suitable carrier molecule, most commonly HDL, which sequesters > 98% of plasma S1P. S1P binds to five cognate high affinity G protein‐coupled receptors to regulate the normal development of arteries, the regulation of blood vessel caliber, the maintenance of endothelial integrity, the normal egress of lymphocytes and their recirculation, uterine implantation of fertilized ova, and hair cell survival *inter alia*. In addition, the immunopathology of certain RNA viruses is quite sensitive to modulation of an S1P signaling rheostat. Experiments on Influenza H1N1 2009, mouse pulmonary virus, and SARS‐1 infections (Oldstone & Rosen, 2014) showed that agonists of the S1P1 receptor suppressed cytokine storm and the IFN‐α auto‐amplification loop protecting from lethality and synergizing with inhibitors of viral replication such as oseltamivir (Teijaro *et al*, 2014). In contrast, S1P1 receptor antagonists exacerbated cytokine storm and lethality (Teijaro *et al*, 2016). The role of S1P signaling tone is tantalizingly framed in the Marfia *et al* (2021) study.

Serum S1P measurements quantify the blood load of S1P, because most biogenesis of S1P occurs through the action of sphingosine kinase‐1 in erythrocytes, followed by egress through carrier channels leading to partitioning with plasma lipids most notably HDL. While the gold standard methodology for the measurement of S1P is liquid chromatography–mass spectrometry, the exigencies of the pandemic required these investigators of test the S1P hypothesis with the practical and feasible ELISA. Each prospective patient sample was significant, because it compared *n* = 111 consecutively hospitalized confirmed COVID‐19 patients from March–May of 2020 at the height of the pandemic’s most difficult phase in Lombardy. This consecutive timing minimized the chances for a selection bias in the study. These patients were compared to 47 healthy subjects.

ApoM levels can be considered a surrogate for the plasma S1P reservoir, that delivers S1P to its receptors, and indeed, Marfia *et al* (2021) show a significant Pearson correlation between S1P and ApoM levels, even though HDL‐C levels were not available for all COVID‐19 patients. HDL‐C had a mean of 59 mg/dl in healthy controls and a range from 47 to 72 mg/dl which contrasted with 36 (28–45) mg/dl for patients admitted with COVID‐19 (*P* < 0.0001). In fact, these populations do not overlap in this study. These data, taken together with the total blood S1P measurements by ELISA, show that S1P ligand concentrations are lower within the hospitalized COVID‐19 infection population compared to healthy controls. The lower levels of S1P ligand (the input) are then susceptible to degradation by cell surface phosphatases and intracellular lyases that further downregulate S1P receptor occupancy and signaling. These data lead to the hypothesis that lower signaling tone from S1P receptors results in increasing susceptibility of interferon‐α auto‐amplification from plasmacytoid dendritic cells and poorer maintenance of endothelial integrity, as well as providing an environment in which small increases in S1P produced by inflammatory processes could result in lymphocyte sequestration and the lymphopenia seen during the COVID‐19 crisis.

Therefore, the broader implication of low HDL and likely low S1P ligand as a predictor of serious COVID‐19 infection is significant (Fig 1). Low HDL is associated with metabolic syndrome, type 2 diabetes, obesity, and lack of physical conditioning. All of these features have been associated with poor outcome from the inflammatory phase of COVID‐19 infection. The hypothesis here is that these metabolic risks have a mechanistic link though S1P and its signaling. Therefore, it is predicted that S1PR modulators that alter S1P signaling tone should be protective against excessive inflammatory amplification of cytokines and the late sequelae of serious COVID‐19 infection. Clinical trials are ongoing in Canada with ozanimod to examine possible therapeutic intervention using this mechanism. It is also possible that the infusion of HDL could replicate this physiology and provide a measure of protection.

**Figure 1:**
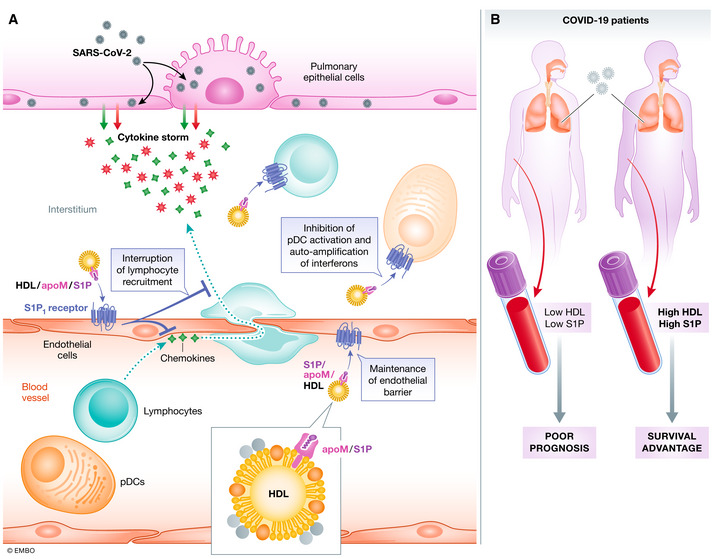
S1P/HDL as a prognostic indicator for ICU entry and mortality of COVID‐19 patients (A) SARS‐CoV‐2 infects pulmonary epithelial cells leading to the upregulation of chemokines and cytokines that prime the recruitment of inflammatory leukocytes including innate inflammatory cells and lymphocytes driving adaptive immunity. The immune collateral damage to lung tissue drives the inflammatory phase and poor outcomes in COVID‐19 patients. Blood S1P, largely transported (> 98%) within HDL, dampens the cytokine storm by interrupting lymphocyte recruitment, activation, and the auto‐amplification of interferon‐α in plasmacytoid dendritic cells by acting on receptors for S1P such as S1PR1, found on all these cell types including endothelium. (B) The risk stratification by Marfia *et al* points to low serum S1P/HDL as a poor prognostic indicator for ICU entry and mortality, whereas high S1P/HDL predicts a better outcome. Increased signaling of the S1P rheostat may present a survival advantage.

The rapid improvement in availability of polymerase inhibitors, such as remdesivir, together with the use of immune sera and monoclonal antibodies provide possibilities for diminishing viral load. Dexamethasone has been shown empirically to provide significant protection from the immunopathology. Therapies that can ameliorate or interrupt the inflammatory phase and cytokine storm such as IL‐6 for example offer helpful strategies though the sequestration of key cytokine mediators may be alternatives, once their mechanisms are better understood and clinically proven.

However, study of the last pandemic provides us with a means of modulating the inflammatory phase of that specific virus. In a world of rapid travel in which pathogens have no real physical boundary between themselves and the large, vulnerable populations, it is imperative to understand the risks that stem from human behaviors such as low HDL and that lead to deleterious immune responses that can favorably modulated using potential tools such as corticosteroids and S1P receptor modulators. These understandings can then be deployed empirically when new and unexpected viral pathogens challenge populations and the health systems that care for them.
